# Supplementary material for: RNAmining: A machine learning stand-alone and web server tool for RNA coding potential prediction
Source: F1000Res. 2021 Jun 8;10:323. Originally published 2021 Apr 26. [Version 2] doi: 10.12688/f1000research.52350.2 (PMC8201426; doi:10.12688/f1000research.52350.2)
Supplement: Peer review history

# Peer review: Reviewer response for version 2

**DOI:** 10.5256/f1000research.57466.r87006

**Published:** 2021-06-11

**Authors:** Gilderlanio Araújo

**Affiliations:** 1 Laboratory of Human and Medical Genetics, Postgraduate Program in Genetics and Molecular Biology, Universidade Federal do Pará, Belém, Brazil

The authors made the corrections. The authors clarified the text and added prediction probabilities to the software.

Are the conclusions about the tool and its performance adequately supported by the findings presented in the article?

Yes

Is the rationale for developing the new software tool clearly explained?

Yes

Is the description of the software tool technically sound?

Yes

Are sufficient details of the code, methods and analysis (if applicable) provided to allow replication of the software development and its use by others?

Yes

Is sufficient information provided to allow interpretation of the expected output datasets and any results generated using the tool?

Partly

Reviewer Expertise:

Bioinformatics. Machine Learning. Transcriptome Analysis. Population Genomics.

I confirm that I have read this submission and believe that I have an appropriate level of expertise to confirm that it is of an acceptable scientific standard.

# Peer review: Reviewer response for version 2

**DOI:** 10.5256/f1000research.57466.r87007

**Published:** 2021-06-09

**Authors:** Andre Yoshiaki Kashiwabara

**Affiliations:** 1 Department of Computer Science, Bioinformatics Graduate Program, Federal University of Technology - Parana, Curitiba, Brazil

The authors have addressed all my concerns.

Are the conclusions about the tool and its performance adequately supported by the findings presented in the article?

Partly

Is the rationale for developing the new software tool clearly explained?

Yes

Is the description of the software tool technically sound?

Yes

Are sufficient details of the code, methods and analysis (if applicable) provided to allow replication of the software development and its use by others?

Yes

Is sufficient information provided to allow interpretation of the expected output datasets and any results generated using the tool?

Yes

Reviewer Expertise:

Bioinformatics, Computational Biology, Machine Learning.

I confirm that I have read this submission and believe that I have an appropriate level of expertise to confirm that it is of an acceptable scientific standard.

# Peer review: Reviewer response for version 1

**DOI:** 10.5256/f1000research.55616.r84936

**Published:** 2021-05-25

**Authors:** Andre Yoshiaki Kashiwabara

**Affiliations:** 1 Department of Computer Science, Bioinformatics Graduate Program, Federal University of Technology - Parana, Curitiba, Brazil

This paper presents RNAmining to predict the protein-coding potential of transcripts. The authors have compared many algorithms using cross-validation and selected XGBoost. The tool has the potential to be very useful. It is available online, and it is easy to use. 

1. In recent studies, some small ORF in annotated ncRNA has validated protein-coding potential ^1^. How does RNAmining behave when these annotated ncRNAs that contain such small ORF challenge it? 
2. It is important to cite RNAploc ^2^, BASINET ^3^, and CoDaN ^4^.
3. I suggest plotting ROC curves when comparing classification methods.
4. In the abstract, the authors have described the use of cross-validation to assess the accuracy of each classification method. However, in methodology, the author explained that 80% is the training set and the other 20% is the validation set. And for CNN, and ANN this number is also different (60%/20%). It isn't clear. It will help create a figure showing the "workflow" of the Training/Validation/Testing part.
5. The standard deviation of the cross-validation can be helpful to show the stability of each classification method. 

Are the conclusions about the tool and its performance adequately supported by the findings presented in the article?

Partly

Is the rationale for developing the new software tool clearly explained?

Yes

Is the description of the software tool technically sound?

Yes

Are sufficient details of the code, methods and analysis (if applicable) provided to allow replication of the software development and its use by others?

Yes

Is sufficient information provided to allow interpretation of the expected output datasets and any results generated using the tool?

Yes

Reviewer Expertise:

Bioinformatics, Computational Biology, Machine Learning.

I confirm that I have read this submission and believe that I have an appropriate level of expertise to confirm that it is of an acceptable scientific standard, however I have significant reservations, as outlined above.

## References

[1] : LncRNA-Encoded Peptide: Functions and Predicting Methods. *Front Oncol* .2020;10: 10.3389/fonc.2020.622294 622294 10.3389/fonc.2020.622294 33520729PMC7842084

[2] : Non-coding transcription characterization and annotation: a guide and web resource for non-coding RNA databases. *RNA Biol* .2012;9(3) : 10.4161/rna.19352 274-82 10.4161/rna.19352 22336709

[3] : BASiNET—BiologicAl Sequences NETwork: a case study on coding and non-coding RNAs identification. *Nucleic Acids Research* .2018;46(16) : 10.1093/nar/gky462 10.1093/nar/gky462 PMC614482729873784

[4] : CodAn: predictive models for precise identification of coding regions in eukaryotic transcripts. *Brief Bioinform* .2021;22(3) : 10.1093/bib/bbaa045 10.1093/bib/bbaa045 32460307PMC8138839

# Response

**Published:** 2021-06-02

**Authors:** Thaís A. R. Ramos

**Affiliations:** Universidade Federal do Rio Grande do Norte, Brazil

1- RNAmining was trained using coding genes and ncRNAs from the Ensembl database. It evaluates the patterns in the tri-nucleotide counts in any RNA sequence (which could be an ORF or not) and, according to this, it classifies into coding and non-coding sequences. The RNAmining training was not performed with the proposal of generating a specific model for ORFs or some specific type of sequence, it works independently of this.

 2- Thank you for your suggestion. We included the citations for BASiNET and CoDaN in the Introduction section of the manuscript. We were not able to find the manuscript describing RNAploc and it was not included.

 3- We believe that it is possible to visualize the performance of the methods from the tables presented along the main text of the paper, as well as made available as supplementary material, since the measures used for the ROC curves construction are the same presented there. In addition, we consider that when we have too similar numbers it is easier to see the difference in a table.

 4- The cross-validation method was used in the grid search method using the training dataset to validate the hyperparameters, choosing the best set of parameters. In addition, this partition method validates the hyperparameter's results through different validation sets. Therefore, it proves that our model is working and generalizing the problem. Thus, when we had the best parameters we used the test dataset (20%) to generate the final models and to calculate the metrics. The connectionist methods (e.g. Artificial Neural Networks and Convolutional Neural Networks) demand a validation dataset to adjust the model, because of the weights optimization stage and its hyperparameters. Thus, for experiments with ANN and CNN, 20% was used for validation, 60% for training (defined as 80% for the other algorithms) and 20% for testing, in all the cases. In addition, due to the complexity of these 2 algorithms, it is more common in literature to use the holdout (training/validation/test) partition method instead of cross-validation. Thereby, we modified the sentence in the abstract: "All the machine learning algorithms tests were performed using 10-folds cross-validation..." to "The machine learning algorithms validations were performed using 10-fold cross-validation...". In addition, in the section "Training and testing datasets, model building and quality measuring for coding potential evaluation" we changed the following sentence: "Sequences were randomly divided into training and testing datasets, using 80% of the data for training and 20% for testing. For ANN and CNN experiments, sequences were split into 60% of the data for training and 20% for validation. The testing dataset was the same used in the other machine learning algorithms." to the following text: "The cross-validation approach was applied in the grid search method, using the training dataset to validate the hyperparameters and obtain the best set of this to be used. In addition, this partition method validates the hyperparameter's results through different validation sets. Therefore, it proves that our model is working and generalizing the problem. Thus, sequences were randomly divided into training and testing datasets, using 80% of the data for training and 20% for testing. The connectionist methods (e.g. Artificial Neural Networks and Convolutional Neural Networks) demand a validation dataset to adjust the model, because of the weights optimization stage and its hyperparameters. Thus, for experiments with ANN and CNN, 20% were used for validation, 60% for training (defined as 80% for the other algorithms) and 20% for testing.The testing dataset was the same used in all machine learning algorithms."

 5- The results shown in this paper are not obtained using cross-validation. The cross-validation method was used in the grid search method to validate the hyperparameters, choosing the best set of parameters. Thus, we can validate the hyperparameter's results through different validation sets. Therefore, it proves that our model is working and generalizing the problem. Thus, to generate the final models and to calculate the metrics, we used the test dataset (20%) with the best parameters.

# Peer review: Reviewer response for version 1

**DOI:** 10.5256/f1000research.55616.r83973

**Published:** 2021-05-10

**Authors:** Gilderlanio Araújo

**Affiliations:** 1 Laboratory of Human and Medical Genetics, Postgraduate Program in Genetics and Molecular Biology, Universidade Federal do Pará, Belém, Brazil

1. Are there two different datasets of model organisms?
  On the abstract "... **15 organisms** from different evolutionary branches..." 
  On the main text "RNAmining was evaluated through **24 organisms** from the eukaryotic tree of life and its results outperformed publicly available tools commonly used for that purpose."
2. Why fine-tuning SVM was performed with a grid search strategy and not Random Forest too? Provide some reasoning.
3. Why sequences were divided using different proportions? Provide some reasoning.
  "Sequences were randomly divided into training and testing datasets, using **80% of the data for training and 20%** for testing. For ANN and CNN experiments, sequences were split into **60% of the data for training and 20% ** for validation."
4. On the web tool, you should provide a column for prediction probability for coding and non-coding variants. The new column will improve user analysis, such as filtering for those predictions with XGboost >= 0.9.

Are the conclusions about the tool and its performance adequately supported by the findings presented in the article?

Yes

Is the rationale for developing the new software tool clearly explained?

Yes

Is the description of the software tool technically sound?

Yes

Are sufficient details of the code, methods and analysis (if applicable) provided to allow replication of the software development and its use by others?

Yes

Is sufficient information provided to allow interpretation of the expected output datasets and any results generated using the tool?

Partly

Reviewer Expertise:

Bioinformatics. Machine Learning. Transcriptome Analysis. Population Genomics.

I confirm that I have read this submission and believe that I have an appropriate level of expertise to confirm that it is of an acceptable scientific standard, however I have significant reservations, as outlined above.

# Response

**Published:** 2021-06-02

**Authors:** Thaís A. R. Ramos

**Affiliations:** Universidade Federal do Rio Grande do Norte, Brazil

1- The connectionist methods (e.g. Artificial Neural Networks and Convolutional Neural Networks) demand a validation dataset to adjust the model, because of the weights optimization stage and its hyperparameters. Thus, for experiments with ANN and CNN, 20% were used for validation, 60% for training (defined as 80% for the other algorithms) and another 20% for testing, in all the cases.

 2- Yes, in fact we have the dataset 1 composed of 15 model organisms which were used to build the models, and the dataset 2 composed of other 9 phylogenetically related and unrelated organisms that were not used in our training, demonstrating the efficiency of the tool even when applied in species phylogenetically distant from those used in training. On the main text, we changed this sentence in the “Introduction” section to: "Next, RNAmining was evaluated in another 9 phylogenetically related and unrelated organisms that were not used in our training, demonstrating the efficiency of the tool even when applied in species phylogenetically distant from those used in training. In total, it was evaluated through 24 organisms from the eukaryotic tree of life and its results outperformed publicly available tools commonly used for that purpose.”

 3- In fact all the methods were executed with the grid search method. We made a mistake in the writing. It was modified by replacing the sentences: "The Random Forest model was implemented using empirical tests and the best result was selected for training the model. We considered the default parameters with the exception of the number of trees used (150 estimators) and the criterion parameter setted to 'entropy' for information gain. KNN and Naive Bayes models were trained with the default values. The SVM parameters were obtained through grid search method and the resulting model was trained with the Radial Basis Function (RBF) kernel, with the Regularization parameter (C) and Kernel coefficient (Gamma) defined in 1000 and 0.8, respectively. ANN and DL were performed with different architectures according to grid search and empirical tests" to the following: "XGBoost, K-NN and Naive Bayes models were trained with the default values. The Random Forest and SVM parameters were obtained through grid search method, the best results using Random Forest resulted in a model generated with the default parameters, with the exception of the number of trees used (150 estimators) and the criterion parameter setted to 'entropy' for information gain. For SVM, the resulting model was trained with the Radial Basis Function (RBF) kernel, with the Regularization parameter (C) and Kernel coefficient (Gamma) defined in 1000 and 0.8, respectively. ANN and DL were performed with different architectures according to grid search and empirical tests."

 4- Thank you for your suggestion. We considered it and provided a new column in the output file (Classification probabilities) in both web server and stand-alone versions.
